# Lessons Learned From a Sequential Mixed-Mode Survey Design to Recruit and Collect Data From Case-Control Study Participants: Formative Evaluation

**DOI:** 10.2196/56218

**Published:** 2024-05-27

**Authors:** Amanda D Tran, Alice E White, Michelle R Torok, Rachel H Jervis, Bernadette A Albanese, Elaine J Scallan Walter

**Affiliations:** 1 Department of Epidemiology Colorado School of Public Health University of Colorado Aurora, CO United States; 2 Colorado Department of Public Health and Environment Denver, CO United States; 3 Adams County Health Department Brighton, CO United States

**Keywords:** case-control studies, mixed-mode design, epidemiologic study methods, web-based survey, telephone interview, public health, outbreak preparedness, COVID-19, survey, recruitment, epidemiology, methods

## Abstract

**Background:**

Sequential mixed-mode surveys using both web-based surveys and telephone interviews are increasingly being used in observational studies and have been shown to have many benefits; however, the application of this survey design has not been evaluated in the context of epidemiological case-control studies.

**Objective:**

In this paper, we discuss the challenges, benefits, and limitations of using a sequential mixed-mode survey design for a case-control study assessing risk factors during the COVID-19 pandemic.

**Methods:**

Colorado adults testing positive for SARS-CoV-2 were randomly selected and matched to those with a negative SARS-CoV-2 test result from March to April 2021. Participants were first contacted by SMS text message to complete a self-administered web-based survey asking about community exposures and behaviors. Those who did not respond were contacted for a telephone interview. We evaluated the representativeness of survey participants to sample populations and compared sociodemographic characteristics, participant responses, and time and resource requirements by survey mode using descriptive statistics and logistic regression models.

**Results:**

Of enrolled case and control participants, most were interviewed by telephone (308/537, 57.4% and 342/648, 52.8%, respectively), with overall enrollment more than doubling after interviewers called nonresponders. Participants identifying as female or White non-Hispanic, residing in urban areas, and not working outside the home were more likely to complete the web-based survey. Telephone participants were more likely than web-based participants to be aged 18-39 years or 60 years and older and reside in areas with lower levels of education, more linguistic isolation, lower income, and more people of color. While there were statistically significant sociodemographic differences noted between web-based and telephone case and control participants and their respective sample pools, participants were more similar to sample pools when web-based and telephone responses were combined. Web-based participants were less likely to report close contact with an individual with COVID-19 (odds ratio [OR] 0.70, 95% CI 0.53-0.94) but more likely to report community exposures, including visiting a grocery store or retail shop (OR 1.55, 95% CI 1.13-2.12), restaurant or cafe or coffee shop (OR 1.52, 95% CI 1.20-1.92), attending a gathering (OR 1.69, 95% CI 1.34-2.15), or sport or sporting event (OR 1.05, 95% CI 1.05-1.88). The web-based survey required an average of 0.03 (SD 0) person-hours per enrolled participant and US $920 in resources, whereas the telephone interview required an average of 5.11 person-hours per enrolled participant and US $70,000 in interviewer wages.

**Conclusions:**

While we still encountered control recruitment challenges noted in other observational studies, the sequential mixed-mode design was an efficient method for recruiting a more representative group of participants for a case-control study with limited impact on data quality and should be considered during public health emergencies when timely and accurate exposure information is needed to inform control measures.

## Introduction

Often used during disease outbreak investigations, case-control studies that retrospectively compare people who have a disease (case participants) with people who do not have the disease (control participants) are an efficient and relatively inexpensive method of identifying potential disease risk factors to guide control measures and interventions. Perhaps the most critical and challenging component of conducting a case-control study is the recruitment of appropriate control participants who are from the same source population as case participants [1]. Because control participants are not ill and may not be connected to the outbreak, they may be less motivated to complete a lengthy questionnaire that collects personal information and detailed exposure histories [2-4]. Moreover, with the increased use of mobile telephones and the routine use of caller ID, study participants contacted by traditional telephone-based survey methodologies may be less likely to answer the telephone [5,6], further reducing the opportunity for participant screening and recruitment.

Recruitment challenges are not unique to case-control studies, and other types of observational studies have shifted from traditional telephone interviews to web-based surveys with the goal of reaching larger groups of people more efficiently and at a lower cost [7-12]. While offering some advantages over traditional telephone interviews, web-based surveys often experience lower response rates and lower data quality [13], and some studies have found demographic differences between telephone and web-based survey participants, likely driven in part by disparities in internet connectivity and access [14]. For this reason, researchers have increasingly used both telephone interviews and web-based surveys in a sequential mixed-mode design, first contacting participants using a self-administered web-based survey, and then following up with nonresponders with an interviewer-administered telephone survey [15]. In other types of observational studies, this mixed-mode design has been shown to reduce selection bias, reduce costs, improve data quality, and result in higher response rates and faster participant recruitment [16,17], making it an appealing design choice for case-control studies.

In March 2020, the World Health Organization declared COVID-19 a global pandemic, and throughout many countries, public health or other governmental authorities implemented stay-at-home orders, travel restrictions, and other public health interventions to reduce disease transmission. In the absence of adequate data-driven evidence about community risk factors for COVID-19 transmission, we implemented a sequential mixed-mode case-control study design in Colorado to evaluate community exposures and behaviors associated with SARS-CoV-2 infection and inform public health control measures. While the benefits and limitations of sequential mixed-mode designs have been well-documented in other contexts [14,16,18-20], they have not been examined in the context of rapidly implemented epidemiological case-control studies. In this paper, we discuss the challenges, benefits, and limitations of using a sequential mixed-mode survey design using web-based surveys disseminated via SMS text message and telephone interviews for a case-control study assessing exposures during a public health emergency. Specific aims are (1) to compare the sociodemographic characteristics of web-based and telephone survey participants, (2) to evaluate the representativeness of survey participants to the sample population, (3) to assess the completeness of participant responses by survey mode, and (4) to estimate the time and resources required to recruit web-based and telephone survey participants.

## Methods

### Case-Control Study Design and Implementation

The case-control study was conducted among Colorado adults aged 18 years and older who had a positive (case) or negative (control) SARS-CoV-2 reverse transcription-polymerase chain reaction test result in Colorado’s electronic laboratory reporting (ELR) system with a specimen collection date from March 16 to April 29, 2021 [21]. Eligible individuals testing positive with a completed routine public health interview in Colorado’s COVID-19 surveillance system were randomly selected and individually matched on age (±10 years), zip code (urban areas) or region (rural and frontier areas), and specimen collection date (±3 days) with up to 20 individuals with a negative test, with the goal of enrolling 2 matched controls per enrolled case.

Self-administered (web-based) and interviewer-administered (telephone) case and control surveys were developed in Research Electronic Data Capture (REDCap; Vanderbilt University). REDCap is a secure, web-based platform designed to support data capture for research studies [22]. The surveys asked about contact with a person with confirmed or suspected COVID-19, travel history, employment, mask use, and community exposure settings (bar or club; church, religious, or spiritual gathering; gathering; grocery or retail shopping; gym or fitness center; health care setting; restaurant, cafe, or coffee shop; salon, spa, or barber; social event; or sports or sporting events) during the 14 days before illness onset or specimen submission. The full survey questionnaire is available in Multimedia Appendix 1. Demographic data were obtained from Colorado’s COVID-19 case surveillance system and the control survey. Web-based surveys were offered in English and Spanish and included clarifying language, prompts, skip logic, text piping, and progress bars. Interviewers used computer-assisted telephone interviewing in REDCap with scripting and language line services when needed. Questions and response options were identically worded in the web-based and telephone surveys, with the exception of a “refused” option for questions in the telephone survey.

Using the Twilio integration in REDCap, selected individuals were sent an SMS text message to the telephone number provided at the time of testing (which may include both landlines and mobile phones) 3 to 7 days after their specimen collection date, inviting them to complete the web-based survey. A team of trained interviewers began contacting nonresponders for telephone interviews approximately 3 hours after the initial SMS text message was sent, making 1 contact attempt for individuals testing positive for SARS-CoV-2 and up to 2 contact attempts for those testing negative. Interviewers only contacted as many controls by telephone as needed to enroll 2 matched controls per enrolled case. The web-based survey link was resent via SMS text message or sent via email when requested. When possible, voicemail messages were left encouraging SMS text message recipients to complete the web-based survey. As the goal of the case-control study was to assess the risk of SARS-CoV-2 infection from community exposures, we only included surveys that had responses to all 15 community exposure questions. Partial surveys that did not have complete community exposure data were excluded from analyses. Individuals were also excluded if they reported living in an institution, close contact with a household member with confirmed or suspected COVID-19, receiving ≥1 dose of a COVID-19 vaccine (which was not universally available in Colorado at the time of the study), symptom onset date >7 days from specimen collection (case participants), a prior positive COVID-19 result (control participants), or providing personal identifying information in the web-based survey that was inconsistent with information from the ELR system (control participants).

### Evaluation of a Sequential Mixed-Mode Survey Design

We evaluated the impact of conducting the COVID-19 case-control study using a sequential mixed-mode design by (1) comparing the sociodemographic characteristics of web-based and telephone survey participants, (2) evaluating the representativeness of study participants to the sample population, (3) assessing the completeness of participant responses by survey mode, and (4) estimating the time and resources required to recruit web-based and telephone survey participants. All analyses were performed using SAS (version 9.4; SAS Institute).

### Comparison of Web-Based and Telephone Survey Participants

Case and control participants were eligible individuals who completed the web-based or telephone survey. We compared the demographic characteristics (age, gender, race and ethnicity, geographic location, working outside the home, and socioeconomic factors) of case and control participants completing the web-based and telephone survey to each other using 2-tailed *t* tests, Pearson *χ*^2^, or Fisher exact tests. Socioeconomic factors, which are not routinely asked in surveillance and therefore not included in the survey, were evaluated by aggregating mean scores for 4 Colorado EnviroScreen indicators (less than high school education, linguistic isolation, low income, and people of color) based on the participant’s county of residence. Colorado EnviroScreen (version 1.0; Colorado State University and the Colorado Department of Public Health and Environment) is a publicly available environmental justice mapping tool developed by the Colorado Department of Public Health and Environment and Colorado State University that evaluates 35 distinct environmental, health, economic, and demographic indicators. Colorado EnviroScreen scores range from 0 to 100, with the highest score representing the highest burden of health injustice.

### Representativeness of Study Participants

We compared the demographic characteristics (as described earlier) of case and control participants completing the web-based and telephone surveys (separately and combined) to the sample pool of all randomly selected individuals testing positive (case sample pool) or negative (control sample pool) for SARS-CoV-2 using 2-tailed *t* tests, Pearson *χ*^2^, or Fisher exact tests.

### Participant Responses

We evaluated data completeness and differential responses between web-based and telephone survey modes by comparing responses to exposure and behavior questions we deemed prone to social desirability bias (close contact with individuals with confirmed or suspected COVID-19, community exposures, travel, and mask use). Two bivariate logistic regression models, the first adjusting for case-control status and the second adjusting for case-control status and sociodemographic variables shown to be associated with mode effects (age, gender, race and ethnicity, and geographic location), examined the association between survey mode and participant response. Question nonresponse, where data were missing or refused, was evaluated for these questions as well as for other questions with free-text or multiple-choice response options (industry, occupation, reasons for COVID-19 testing, and mask type).

### Time and Resource Needs

The time spent by study personnel contacting potential participants by SMS text message and telephone was obtained from self-recorded data in timesheets and used to calculate the person-hours required per enrolled participant. Total expenditures for the web-based and telephone surveys were calculated using staff wages and Twilio texting costs (an average of US $0.008 for a 160-character SMS text message).

### Ethical Considerations

The case-control study was deemed by the Colorado Multiple Institutional Review Board to be public health surveillance and not human participant research and was therefore exempt from full approval and requirements for informed consent (protocol 21-2973).

## Results

### Case and Control Participant Enrollment

**Figure 1:**
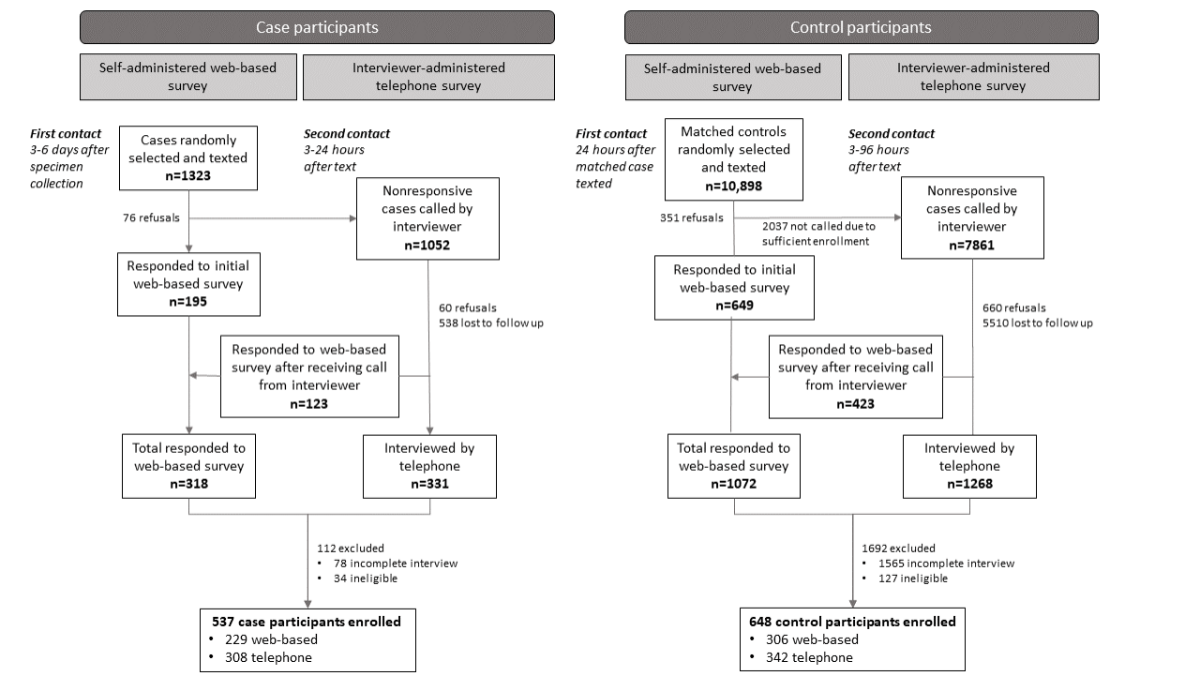
Web-based and telephone survey enrollment among case and control participants, Colorado, March to April 2021.

The case sample pool included 1323 individuals. Of these, 318 (24%) responded to the web-based survey, and 331 (25%) were interviewed by telephone (Figure 1). A total of 537 (40.6%) case participants were enrolled after excluding 78 (5.9%) partial and 34 (2.6%) ineligible survey responses. Of the 10,898 individuals in the control sample pool, 1072 (9.8%) responded to the web-based survey, and 1268 (11.6%) were interviewed by telephone. A total of 648 (5.9%) control participants were enrolled after excluding 1565 (14.4%) partial and 127 (1.2%) ineligible surveys. Of the enrolled case and control participants, most were interviewed by telephone (308/537, 57.4% and 342/648, 52.8%, respectively).

### Comparison of Web-Based and Telephone Survey Participants

Case participants completing the web-based and telephone surveys were similar in age (mean 37, SD 13.21 and 14.69 years, respectively), whereas web-based control participants were slightly older than those completing the telephone survey (mean 38, SD 12.44 vs mean 36, SD 12.62 years, respectively; Table 1). For both case and control participants, those aged 40-59 years were more likely to complete the web-based survey, whereas participants aged 18-39 years and 60 years and older were more likely to complete the telephone survey. Web-based case and control participants were more likely to identify as female, White, non-Hispanic, reside in urban areas, and be less likely to work outside the home. Compared to web-based case and control participants, telephone participants had higher EnviroScreen scores for all socioeconomic indicators, indicating they resided in counties with larger populations of individuals with less than high school education, linguistic isolation, low income, and people of color.

**Table 1:** Comparison of sociodemographic characteristics of the case and control sample pools with case and control participants overall and by survey completion mode, Colorado, March to April 2021.

Characteristic		Individuals with a positive SARS-CoV-2 test result				Individuals with a negative SARS-CoV-2 test result			
		Case participant			Case sample pool^a^ (n=1323)	Control participant			Control sample pool^b^ (n=10,898)
		Web-based (n=229)	Telephone (n=308)	Total enrolled (n=537)		Web-based (n=306)	Telephone (n=342)	Total enrolled (n=648)	
Age (years), mean (SD)^c^		37 (13.21)	37 (14.69)	37 (14.06)	37 (14.20)	38 (12.44)	36 (12.62)^d^	37 (12.60)	38 (13.40)
**Age group (years), n (%)^c,e^**									
	18-29	75 (32.8)	114 (37)	189 (35.2)	478 (36.1)	81 (26.5)^f^	119 (34.8)	200 (30.9)	3385 (31.1)
	30-39	57 (24.9)	77 (25)	134 (25)	322 (24.3)	87 (28.4)^f^	114 (33.3)	201 (31)	3120 (28.6)
	40-49	49 (21.4)	47 (15.3)	96 (17.9)	234 (17.7)	74 (24.2)^f^	55 (16.1)	129 (19.9)	2107 (19.3)
	50-59	35 (15.3)	37 (12)	72 (13.4)	174 (13.2)	51 (16.7)^f^	35 (10.2)	86 (13.3)	1411 (13)
	≥60	13 (5.7)	33 (10.7)	46 (8.6)	115 (8.7)	13 (4.3)^f^	19 (5.6)	32 (4.9)	875 (8)
**Sex, n (%)^c,e,g^**									
	Female	134 (58.8)^f^	149 (48.5)	283 (52.9)	642 (48.7)	172 (57)	146 (52.3)	318 (54.7)	—^h^
	Male	94 (41.2)^f^	157 (51.1)	251 (46.9)	675 (51.2)	125 (41.4)	131 (47)	256 (44.1)	—
	Another sex	0 (0)^f^	1 (0.3)	1 (0.2)	1 (0.1)	5 (1.6)	2 (0.7)	7 (1.2)	—
	Missing or unknown	1 (0.4)^f^	1 (0.3)	2 (0.4)	5 (0.4)	4 (1.3)	63 (18.4)	67 (10.3)	—
**Race and ethnicity, n (%)^e^**									
	Black, non-Hispanic	3 (1.5)	9 (3.4)	12 (2.6)	35 (3.1)	6 (2.3)^d^	13 (4.9)^d^	19 (3.6)	341 (4.4)
	Hispanic (any race)	44 (21.9)	91 (34.6)	135 (29.1)	330 (28.9)	45 (16.9)^d^	59 (22)^d^	104 (19.4)	1117 (14.3)
	White, non-Hispanic	146 (72.6)	148 (56.3)	294 (63.4)	722 (63.2)	205 (76.8)^d^	180 (67.2)^d^	385 (72)	4467 (57.2)
	Another race or ethnicity	8 (4)	15 (5.7)	23 (5)	55 (4.8)	11 (4.1)^d^	16 (6)^d^	27 (5.1)	1887 (24.2)
	Missing or unknown	28 (12.2)	45 (14.6)	73 (13.6)	181 (13.7)	39 (12.8)^d^	74 (21.6)^d^	113 (17.4)	3086 (28.3)
**Geographic location, n (%)^c^**									
	Rural or frontier	64 (28)	92 (29.9)	156 (29.1)	386 (29.2)	59 (19.3)^d^	91 (26.6)	150 (23.2)^f^	3057 (28.1)
	Urban	165 (72.1)	216 (70.1)	381 (71)	937 (70.8)	247 (80.7)^d^	251 (73.4)	498 (76.9)^f^	7841 (71.9)
**Work outside the home, n (%)^c,e,g^**		124 (54.1)	201 (65.3)^f^	325 (62.7)	764 (60.3)	147 (48)	188 (56.1)	335 (52.3)	—^h^
	Missing or unknown	9 (3.9)	10 (3.3)	19 (3.5)	55 (4.2)	0 (0)	7 (2.1)	7 (1.1)	—
**EnviroScreen indicator score,^i^ mean (SD)**									
	Less than high school education^c^	47.9 (27.9)	53.2 (28.8)	50.9 (28.5)	51 (28.4)	47.6 (27.7)^d^	55.7 (29)	51.9 (28.7)	53 (29)
	Linguistic isolation^c,e^	60.7 (25.5)	61.4 (26.1)	61.1 (25.8)	61 (25.5)	62.7 (21.6)	64.5 (23.9)	63.6 (22.8)	62.4 (24.6)
	Low income^e^	25.7 (18.4)^d^	31.4 (21.4)	29 (20.3)	29.7 (20.3)	24.7 (21.1)^d^	31.1 (21.5)	28.2 (19.9)^f^	30 (21.1)
	People of color^e^	63.7 (23.6)	65.9 (26)	65 (25)	64.7 (24.8)	65.3 (23)	68.5 (23.7)	66.9 (23.5)	66.6 (24.4)

^a^Individuals with a positive SARS-CoV-2 test result.

^b^Individuals with a negative SARS-CoV-2 test result.

^c^*P*<.05; control participant web-based versus telephone.

^d^*P*<.01; survey mode (web-based, telephone, and web-based and telephone combined) versus sample pool.

^e^*P*<.05; case participant web-based versus telephone.

^f^*P*<.05; survey mode (web-based, telephone, and web-based and telephone combined) versus sample pool.

^g^Information on sex and working outside the home were not available from Colorado’s electronic laboratory reporting system for control participants.

^h^Not available.

^i^Colorado EnviroScreen is an environmental justice mapping tool. Scores are assigned at the county level, with a higher score indicating that an area is more likely to be affected by the indicated health injustice.

### Representativeness of Study Participants

There were statistically significant sociodemographic differences noted between web-based and telephone case and control participants and their respective sample pools (Table 1). More web-based case participants identified as female (134/228, 58.8%) than those in the case sample pool (642/1318, 48.7%). More web-based control participants identified as White, non-Hispanic (205/267, 76.8%) than those in the control sample pool (4467/7812, 57.2%) and more often resided in urban areas (247/306, 80.7%) than those in the control sample pool (7841/10,898, 71.9%). Case and control participants were more similar to their respective sample pools when evaluated as a single group (total enrolled).

### Participant Responses

In the model adjusting for case or control status only, web-based participants were less likely to report close contact with an individual with COVID-19 when compared to telephone participants (odds ratio [OR] 0.70, 95% CI 0.53-0.94) but more likely to report community exposures including visiting a grocery store or retail shop (OR 1.55, 95% CI 1.13-2.12), visiting a restaurant or cafe or coffee shop (OR 1.52, 95% CI 1.20-1.92), attending a gathering outside the home (OR 1.69, 95% CI 1.34-2.15), or attending or participating in a sport or sporting event (OR 1.05, 95% CI 1.05-1.88) in 14 days before symptom onset or specimen collection (Table 2). When adjusted for case or control status, age, gender, race and ethnicity, and geographic location, the only associations that remained statistically significant were close contact (adjusted OR 0.65, 95% CI 0.48-0.88) and gatherings (adjusted OR 1.44, 95% CI 1.12-1.85).

**Table 2:** Association between question response and survey completion mode (web-based vs telephone), Colorado, March-April 2021.

Survey question^a^		Web-based (n=535)		Telephone (n=650)		Telephone as reference,^b^ OR^c^ (95% CI)	Telephone as reference,^d^ adjusted OR (95% CI)
		Responded “Yes,” n (%)	Missing, n (%)	Responded “Yes,” n (%)	Missing or refused, n (%)		
Close contact with individual with confirmed or suspected COVID-19		92 (17.2)	1 (0.2)	150 (23.3)	3 (0.5)	0.70 (0.53-0.94)	0.65 (0.48-0.88)
**Community exposure**							
	Grocery or retail shopping	456 (86)	5 (0.9)	509 (79.5)	10 (1.5)	1.55 (1.13-2.12)	1.12 (0.79-1.57)
	Restaurant, cafe, or coffee shop	256 (49)	13 (2.4)	248 (38.5)	6 (0.9)	1.52 (1.20-1.92)	1.27 (0.99-1.63)
	Gatherings	238 (45)	6 (1.1)	209 (32.5)	3 (0.5)	1.69 (1.34-2.15)	1.44 (1.12-1.85)
	Health care setting	124 (23.3)	2 (0.4)	120 (18.6)	3 (0.5)	1.30 (0.98-1.73)	1.18 (0.88-1.59)
	Sports or sporting event	118 (22.1)	0 (0)	107 (16.7)	2 (0.3)	1.40 (1.05-1.88)	1.32 (0.97-1.80)
	Gym or fitness center	78 (14.7)	3 (0.6)	97 (15.1)	3 (0.5)	0.97 (0.70-1.34)	0.89 (0.64-1.25)
	Salon, spa, or barber	89 (16.7)	2 (0.4)	86 (13.5)	6 (0.9)	1.25 (0.91-1.73)	1.18 (0.84-1.65)
	Bar or club	61 (11.6)	8 (1.5)	71 (11.1)	4 (0.6)	1.05 (0.73-1.51)	0.94 (0.64-1.36)
	Church, religious, spiritual gathering	47 (8.8)	2 (0.4)	58 (9.1)	4 (0.6)	0.98 (0.65-1.46)	1.01 (0.66-1.54)
	Social event	41 (7.7)	2 (0.4)	50 (7.8)	3 (0.5)	0.97 (0.63-1.50)	0.94 (0.64-1.36)
**Travel**		188 (35.7)	9 (1.7)	227 (35.5)	6 (0.9)	0.99 (0.78-1.26)	0.97 (0.75-1.26)
	Airplane or airport	73 (13.6)	1 (0.2)	73 (11.2)	6 (0.9)	1.20 (0.85-1.71)	1.12 (0.78-1.60)
	Hotel	77 (14.4)	1 (0.2)	82 (12.6)	5 (0.8)	1.12 (0.80-1.57)	1.09 (0.77-1.54)
	Public transportation	33 (6.2)	2 (0.4)	52 (8)	8 (1.2)	0.74 (0.47-1.16)	0.77 (0.48-1.23)
**Frequency of indoor mask use**		N/A^e^	1 (0.2)	N/A	9 (1.4)	N/A	N/A
	Always	457 (85.4)	N/A	553 (86.1)	N/A	1.02 (0.74-1.42)	0.87 (0.61-1.23)
	Sometimes	61 (11.4)	N/A	71 (11.1)	N/A	1.06 (0.74-1.52)	1.24 (0.84-1.82)
	Rarely	9 (1.7)	N/A	10 (1.1)	N/A	1.11 (0.45-2.76)	1.11 (0.42-2.90)
	Never	7 (1.3)	N/A	1 (0.2)	N/A	1.17 (0.41-3.35)	1.15 (0.38-3.49)
Industry (free response)		N/A	22 (4.1)	N/A	30 (4.6)	N/A	N/A
Occupation or job title (free response)		N/A	17 (3.2)	N/A	34 (5.2)	N/A	N/A
Reasons tested for COVID-19 (select all reasons)		N/A	18 (3.4)	N/A	2 (0.3)	N/A	N/A
Mask types typically worn (select all types)		N/A	6 (1.1)	N/A	9 (1.4)	N/A	N/A

^a^Full survey questions are available in Multimedia Appendix 1.

^b^Adjusted for case or control status.

^c^OR: odds ratio.

^d^Adjusted for case or control status, age, gender, race and ethnicity, and geographic location.

^e^N/A: not applicable.

Question nonresponse was low across both modalities, with similar ranges of missingness between the web-based survey (0/535, 0% to 22/535, 4.1%) and telephone survey (2/650, 0.3% to 34/650, 5.2%). Nonresponse to industry, occupation, and masking questions was higher in the telephone survey (9/650, 1.4% to 34/650, 5.2%) than the web-based survey (1/535, 0.2% to 22/535, 4.1%; Table 2).

### Time and Resource Needs

Over the course of the study, staff spent a cumulative 15 hours randomly selecting and texting potential participants for the web-based survey, averaging 0.03 person-hours per enrolled participant (15 person-hours per 535 web-based participants) and US $500 in staff wages. Twilio texting costs were US $420, amounting to US $920 in total expenditures for the web-based survey. Comparatively, 3319 hours were spent by interviewers attempting to contact nonresponders by telephone, for an average of 5.11 person-hours per enrolled participant (3319 person-hours per 650 telephone participants) and US $70,000 in interviewer wages.

## Discussion

### Principal Findings

While the web-based survey was more time- and cost-efficient than the telephone interview, participant enrollment was low, and there were statistically significant sociodemographic differences between the web-based case and control participants and their respective sample pools. Adding the follow-up telephone interview increased participant enrollment and the representativeness of both the case and control participants to sample pools. Participant responses to exposure and behavior questions and data completeness were similar between the 2 survey modalities.

Enrollment more than doubled for case and control participants after interviewers called individuals who did not respond to the web-based survey to complete the survey by telephone. Case participant enrollment for our mixed-mode study was higher than those for other COVID-19 case-control studies using telephone only (40.6% vs 3%-25% case participant enrollment in other studies), but control participant enrollment was lower (5.9% vs 9%-13% control participant enrollment in other studies) [23-25]. However, control participant enrollment in our sequential mixed-mode study may not be comparable to telephone-only COVID-19 case-control studies for 2 reasons. First, we texted up to 20 potential controls for every enrolled case participant in anticipation of lower response rates for the web-based survey, inflating the number of contacted controls in our response rate calculations. Second, we did not follow up with all potential controls by telephone once our quota of 2 controls per case was reached. In contrast, telephone-only studies only call as many controls as needed to enroll the desired number of matched control participants, which is typically less than 20.

We found sociodemographic differences between participants completing the survey on the web and by telephone. Web-based respondents were more likely to be female, identify as White, non-Hispanic, have higher levels of education, and reside in urban areas, which was consistent with other studies evaluating survey mode effects [12,26,27]. Contrary to other studies that found higher web-based response rates among those younger than 35 years of age [14], participants aged 18-39 years in our case-control study were more likely to respond to the telephone survey, as were participants aged 60 years and older, participants working outside the home, and participants residing in areas with a higher burden of health injustices. Some of these differences may be attributable to the timing of when potential participants were contacted. While potential participants were texted a link to complete the web-based survey only in the morning, telephone interviews were administered throughout the day, including in the late afternoon and evening when more people may be at home and not working. In addition, older participants and participants in lower socioeconomic settings may experience more barriers to completing a web-based survey, such as limited internet access or less comfort using mobile platforms [15], making them more likely to complete a telephone interview.

While there were sociodemographic differences between web-based and telephone participants and between web-based and telephone case and control participants and their respective sample pools, the sociodemographic characteristics of combined web-based and telephone survey participants were broadly representative of the sample pools. This indicates that the sequential mixed-mode design allowed for the recruitment of more representative case and control participant groups than if we had used a telephone or web-based survey alone, and the use of this survey design can help reduce selection bias in case-control studies.

Telephone surveys conducted by trained interviewers have several advantages over other modes of administration. Most importantly, trained interviewers can answer participants’ questions, add clarifying questions, and probe interviewees for more complete responses, leading to better data completeness and quality. While increasing data quality, telephone surveys can lead to social desirability bias as participants may alter answers to questions to seem more favorable or socially acceptable to an interviewer [19,20]. An advantage of using a web-based survey is that the absence of an interviewer may provide participants with the opportunity to answer questions more candidly, potentially reducing social desirability bias [19,20]. While we found that web-based participants were more likely to report certain community exposures, most of the differential responses between web-based and telephone participants were no longer statistically significant after adjusting for variables shown to be associated with mode effects (age, gender, race, ethnicity, and geographic location). This suggests that demographic differences between web-based and telephone participants may be confounding variables and should be considered when analyzing and interpreting data for case-control studies.

### Limitations

This project was subject to several limitations. First, cases were randomly selected from persons reported in Colorado’s COVID-19 surveillance system who had already completed an interview with public health, which may impact study findings. For example, this method of case-participant selection may account for the high enrollment rates we had for our case-control study, and these individuals may systematically differ from those testing positive for SARS-CoV-2 who did not complete an initial interview with public health. Second, sample pool data were obtained from the ELR system for control participants, which had incomplete demographic data. The sample pool characteristics presented in this paper may not be accurate because of these missing data and, in turn, affect our evaluations of sample representativeness. Third, the socioeconomic characteristics of participants may be subject to ecological fallacy as we used county-level Colorado EnviroScreen scores as a proxy for individual socioeconomic status. Fourth, it is unclear whether the systematic differences noted between web-based and telephone participants were due to the survey mode itself or due to the additional contact attempts made to enroll telephone participants. Finally, this sequential mixed-mode case-control study was implemented during the COVID-19 pandemic, a period marked by various political and social factors that could have influenced who responded to our survey and their responses. As such, findings from this paper may not be generalizable to case-control studies evaluating other diseases or outbreaks.

### Conclusions

Telephone interviews conducted as part of an outbreak investigation are time-consuming and costly [8]. Given the limited resources and staff at many public health agencies, it is critical to find methods to increase efficiency and reduce the costs of outbreak investigations. Web-based surveys are more time- and cost-efficient than telephone interviews, greatly reducing the workload for health departments. However, web-based surveys may appeal to specific demographics, have lower enrollment rates, and may require a larger sample pool or a longer time to enroll participants, which may not be feasible for small outbreaks or ideal for public health emergencies when timely data collection is crucial.

By using a sequential mixed-mode design, we were able to efficiently recruit participants for a case-control study with limited impact on data quality. Moreover, using the sequential mixed-mode approach allowed for maximal sample representativeness compared to a web-based or telephone interview alone. This is critical during public health emergencies, when timely and accurate exposure information is needed to inform control measures and policy. While the sequential mixed-mode design allowed us to reach more potential control participants with fewer resources, we still encountered the same challenges recruiting control participants noted in other studies.

## Appendix

Multimedia Appendix 1 Colorado COVID-19 case-control survey questionnaires.

## Abbreviations

ELR: electronic laboratory reporting
OR: odds ratio
REDCap: Research Electronic Data Capture

## Data Availability

The data sets generated and analyzed during this study are not publicly available due to Colorado state statutes and regulations, which limit data release based on maintaining confidentiality for potentially identifiable person-level data, but are available from the Colorado Department of Public Health and Environment upon reasonable request.
