# Supplementary material for: Lessons Learned From a Sequential Mixed-Mode Survey Design to Recruit and Collect Data From Case-Control Study Participants: Formative Evaluation
Source: JMIR Form Res. 2024 May 27;8:e56218. doi: 10.2196/56218 (PMC11165283; doi:10.2196/56218)
Supplement: Multimedia Appendix 1 [file formative_v8i1e56218_app1.pdf]

**COVID-19 Case-Control Study Questionnaire**
Colorado Department of Public Health and Environment
Colorado School of Public Health

# Case Interview

The Colorado Department of Public Health and Environment and Colorado School of Public Health are sending this survey to people to better understand why some people are getting COVID-19 and others are not. This information will help us better control COVID-19 and direct our public health response so that it meets the needs of communities that are being most affected by COVID-19.

## Before the Call

Complete the following information before calling the case (enter exactly as it appears in the case log):

Your name: ______________________

Case phone number: ______________________

Case name: ______________________

Add any issues or notes you had during this interview here: ______________________

## Participation

Voicemail Script: [interviewer note: If the voicemail is not in English or if the participant prefers another language,please refer to the Language Line instructions and FAQ document. Language Line #: x-xxx-xxx-xxxx (Account:xxxxx)]

"Hi, this message is for [name]. This is [interviewername] from the Colorado Department of Public Health and Environment. You should have gotten a text message earlier to see if you are interested in participating in a short survey about COVID-19 in Colorado. You can still complete the survey via the link in the text message we previously sent you [for Spanish speakers: and that survey link will allow you to choose the Spanish language option]. We would really appreciate your time to help us with this brief survey. If you have any questions, you can call us at (xxx) xxx-xxxx for more information. Thank you for your time, we hope to see your response, and take care."

Interviewer Note: Phone number sending text messages is (xxx) xxx-xxxx

Check this box if left voicemail or unable to contact control — ☐ Voicemail or unable to contact

Check this box if phone number invalid/disconnected — ☐ Invalid or disconnected

Phone Script:

Hi. This is [interviewername] from the Colorado Department of Public Health. May I please speak with [name]?

The Colorado Department of Public Health and Environment is hoping to collect some information to help us better understand why some individuals are becoming ill with COVID-19 and others are not to help direct our public health response throughout the state. I'm giving you a call because you were recently interviewed about a positive COVID-19 test, and we have a few follow up questions that will take about 5 minutes. Do you have time now?

[If they say yes and are curious about what the study is] Thank you, this information will help us better control COVID-19 and direct our public health response so that it meets the needs of communities that are being most affected by COVID-19. Any information you provide us will be confidential and stored in a secure location.

[If they hesitate or say it is not a good time] "Would you be willing to take the survey online on your own time? You should have received a text message with a link, or I can re-send it to you? Is this a good phone number to text, or I can email?" [if yes, insert details below]

What questions or concerns do you have before we continue? If there is anything you don't understand or want me to explain, please let me know at any time during this call.

---

Phone or email for re-sending link: ______________________

---

Check this box if they requested that the link be resent — ☐ Requested that link be resent

---

Check this box if they requested link be resent in Spanish: — ☐ Requested link be resent in Spanish

---

Are you willing to participate in this survey and answer all questions as honestly and accurately as possible?

- ◯ Yes
- ◯ No

---

Do you prefer proceeding in English or in another language?

- ◯ I prefer English.
- ◯ I prefer another language.

---

What language do you prefer? ______________________

---

Interviewer Note: If the case-patient prefers a language other than English, respond with: "Thank you for letting me know. We can use an interpreter to help us complete this interview in [language_other]. I'll go ahead and end this call, and callback with an interpreter on the line, does that sound ok?"

---

What date were you most recently tested for COVID-19? ______________________

This is the date you received the test (had the swab done), not the date you got results.

---

What is the first date you experienced any symptoms? ______________________

---

Check here if interviewee refused these questions: — ☐ Refused

## Exposures and Activities

For this next set of questions, it may be helpful for you to have a calendar in front of you in order to review some dates with me. It is really important for us to be as accurate as possible with these dates. I'd like to ask you some questions about what you did in the 14 days before you started feeling symptoms ([onsetdate]). Feel free to use your calendar, phone, or social media to help jog your memory about what you did and who you had contact with.

---

Did you visit a healthcare setting in the 14 days before you started feeling symptoms?

A healthcare setting could include a doctor's or dentist's appointment, hospital visit, or visit for medical care, either if you were a patient or you visited someone else or went to someone else's appointment with them.

- ◯ Yes
- ◯ No
- ◯ Unknown

---

How many times did you visit a healthcare setting in the 14 days before you started feeling symptoms?

__________________________________

---

As far as you are aware, did you have contact with anyone with confirmed or suspected COVID-19 in the 14 days before you started feeling symptoms?

Contact is defined as being within 6 feet of an infected person for a total of 15 minutes or more, whether they were wearing a mask or not.

Interviewer note: This is only contact that someone is aware of. Many people may say they do not know if they were in contact with someone who had COVID-19, so an unkonwn response here would be "No".

- ◯ Yes
- ◯ No

---

Describe your contact with the known case of COVID-19: Select all that apply.

Congregate housing refers to housing where individuals are living in close quarters or share common spaces

- ☐ Living in the same household
- ☐ In my house but not LIVING in my house (intimate partner, providing care at home)
- ☐ In a healthcare facility (for example, hospital, clinic, MD office, dialysis)
- ☐ In a long-term care facility (for example, skilled nursing facility, rehabilitation center)
- ☐ In other congregate housing / multi-family dwelling (for example, living in close quarters or sharing common spaces)
- ☐ At a daycare or school
- ☐ At my place of employment
- ☐ At a social event or gathering
- ☐ Place of worship / Religious event
- ☐ Political Rally / Gathering
- ☐ Sports Event
- ☐ Travel
- ☐ Unknown/Unsure
- ☐ Other (specify): ______

| | |
|---|---|
| What is your relationship to the person who had (or may have had) COVID-19? Select all that apply. | ☐ Spouse/Partner<br>☐ Child<br>☐ Parent<br>☐ Other Family<br>☐ Friend<br>☐ Healthcare Worker<br>☐ Co-worker<br>☐ Classmate<br>☐ Roommate / housemate<br>☐ Contact only - no relationship<br>☐ Other (specify): ______ |
| As far as you know, did this person test positive for COVID-19? | ◯ Tested positive<br>◯ Tested negative or unsure of results<br>◯ Unsure if tested<br>◯ Not tested |

Now I'd like you to walk me through what you did in the 14 days before you started feeling symptoms ([onsetdate]). For these questions, do not include activities you did as part of paid work. Interviewer note: These questions are intended to capture high-risk exposures, where masking, distancing, and avoiding congregation outside the household may not have been possible.

| | |
|---|---|
| Did you attend any gatherings in the 14 days before you started to feel symptoms?<br><br>Interviewer note: A gathering includes any gathering outside the home (in a public or private setting) with members outside of the individual's household. | ◯ Yes ◯ No ◯ Unknown |
| How many times? | ______________________ |
| Did you go to a restaurant/cafe/coffee shop in the 14 days before you started feeling symptoms (do not include takeout, curbside/window pickup or food delivery)? | ◯ Yes ◯ No ◯ Unknown |
| How many times? | ______________________ |
| How many times did you dine indoors? | ______________________ |
| How many times did you dine in a structure (for example, tents, yurts, igloos, pods, etc) with people not in your household? | ______________________ |
| How many times did you dine outside (open air, not in a structure) with people not in your household? | ______________________ |
| Did you go to a bar or club in the 14 days before you started feeling symptoms? | ◯ Yes ◯ No ◯ Unknown |
| How many times? | ______________________ |
| How many times did you go to a bar or club indoors? | ______________________ |

| | |
|---|---|
| Did you attend any church/religious/spiritual gatherings in the 14 days before you started feeling symptoms? | ◯ Yes ◯ No ◯ Unknown |
| How many times? | __________________ |
| How many times were you indoors at church/religious/spiritual gatherings? | __________________ |
| Did you go to a gym/fitness center/workout group in the 14 days before you started feeling symptoms? | ◯ Yes ◯ No ◯ Unknown |
| How many times? | __________________ |
| How many times were you indoors at a gym/fitness center/workout group? | __________________ |
| Did you attend a large sporting event, such as a Rockies game, in the 14 days before you started feeling symptoms?<br><br>Interviewer note: This includes large, mostly professional events, or sporting events where you pay a fee to enter and attend (as opposed to attending a child or relative's soccer tournament, for example). | ◯ Yes ◯ No ◯ Unknown |
| How many times? | __________________ |
| How many times were you indoors at a sporting event? | __________________ |
| Did you attend a sports practice/game/tournament as a participant or spectator in the 14 days before you started feeling symptoms? | ◯ Yes ◯ No ◯ Unknown |
| How many times? | __________________ |
| How many times were you indoors at a sports practice/game/tournament? | __________________ |
| Were you a: | ☐ Spectator<br>☐ Participant (including player, coach, referee) |
| Did you participate in any snow sports (skiing, snowboarding) in the 14 days before you started feeling symptoms? | ◯ Yes ◯ No ◯ Unknown |
| How many times? | __________________ |
| How many times were you at a resort? | __________________ |

| | |
|---|---|
| Did you attend a wedding/funeral/birthday party (or other similar gathering) in the 14 days before you started feeling symptoms? | ◯ Yes ◯ No ◯ Unknown |
| How many times? | __________________ |
| How many times were you indoors at a wedding/funeral/birthday party, or other similar gathering? | __________________ |
| Did you go to a grocery store (not curbside/outside pickup) in the 14 days before you started feeling symptoms? | ◯ Yes ◯ No ◯ Unknown |
| How many times? | __________________ |
| Did you go to any retail shopping venues other than a grocery store (not curbside/outside pickup) In the 14 days before you started feeling symptoms? | ◯ Yes ◯ No ◯ Unknown |
| How many times? | __________________ |
| Did you go to any protests or rallies in the 14 days before you started feeling symptoms? | ◯ Yes ◯ No ◯ Unknown |
| How many times? | __________________ |
| Did you go to a salon, spa, or barber in the 14 days before you started feeling symptoms?<br><br>Interviewer note: Include hair salons, nail salons, massage, and other similar venues. | ◯ Yes ◯ No ◯ Unknown |
| How many times? | __________________ |
| Did you visit a casino in the 14 days before you started feeling symptoms? | ◯ Yes ◯ No ◯ Unknown |
| How many times? | __________________ |
| Are there any additional places you went or activities you did outside your home in the 14 days before you started feeling symptoms ([onsetdate])? | __________________ |
| Check here if interviewee refused these questions: | ☐ Refused |

## Travel Information

The next few questions I'll ask you are about travel in the 14 days before you started feeling symptoms ([onsetdate]).

| | |
|---|---|
| Did you travel in the 14 days before you started feeling symptoms? Select all that apply. | ☐ International<br>☐ Domestic (within the U.S.)<br>☐ Within Colorado (Specifically, outside of county of residence for work, shopping, or recreation)<br>☐ None |
| Did you spend time on an airplane or in an airport in the 14 days before you started feeling symptoms? | ◯ Yes<br>◯ No |
| Did you stay overnight in a hotel in the 14 days before you started feeling symptoms? | ◯ Yes<br>◯ No |
| Did you use public transportation in the 14 day before you started feeling symptoms?<br><br>Interviewer Note: this does not include ride-share use like Uber or Lyft. | ◯ Yes<br>◯ No |
| How many times? | ______________________ |
| Check here if interviewee refused these questions: | ☐ Refused |

## Mask use and other behaviors

These final questions ask about wearing masks and other behaviors in the 14 days before you started feeling symptoms (remember, you started feeling symptoms on [onsetdate]).

| | |
|---|---|
| When you left your home in the 14 days before you started feeling symptoms, how often did you wear a face covering or mask when in an indoor space? | ◯ Always<br>◯ Sometimes<br>◯ Rarely<br>◯ Never<br>◯ Unknown |
| When you left your home in the 14 days before you started feeling symptoms, how often did you wear a face covering or mask when in an outdoor space? | ◯ Always<br>◯ Sometimes<br>◯ Rarely<br>◯ Never<br>◯ Unknown |
| Why type of mask did you typically wear? | ☐ Cloth mask<br>☐ Medical mask<br>☐ KN95 mask<br>☐ Gaiter<br>☐ Double mask<br>☐ Did not typically wear a mask |

| | |
|---|---|
| Did you take any of the following actions in the 14 days before you started feeling symptoms? | ☐ Social distancing more than 6 feet away from people when outside your home<br>☐ Stayed home almost the entire time<br>☐ Did not visit family or friends inside<br>☐ Did not travel other than local errands<br>☐ Postponed/canceled non-essential medical appointments, procedures, or surgeries<br>☐ Kept others out of your home<br>☐ Increased hand washing or use of hand sanitizer<br>☐ Other: (specify) ______<br>☐ None of the above |

Thank you for participating in this survey! You are helping Colorado understand more about COVID-19 and reduce illness in our state. If you have any questions, please contact the Project Coordinator at xxxxx@state.co.us, call us at (xxx) xxx-xxxx, or visit https://covid19.colorado.gov/case-control-comparison.

| | |
|---|---|
| Add any issues or notes your had during this interviewer here: | __________________________________ |

**COVID-19 Case-Control Study Questionnaire**
Colorado Department of Public Health and Environment
Colorado School of Public Health

# Control Interview

The Colorado Department of Public Health and Environment and Colorado School of Public Health are sending this survey to people to better understand why some people are getting COVID-19 and others are not. This information will help us better control COVID-19 and direct our public health response so that it meets the needs of communities that are being most affected by COVID-19.

## Before the Call

Complete the following information before calling the control (enter exactly as it appears in the control log):

Your name: ______________________

Control phone number: ______________________

Control name: ______________________

Add any issues or notes you had during this interview here: ______________________

## Participation

Voicemail Script: [interviewer note: If the voicemail is not in English or if the participant prefers another language, please refer to the Language Line instructions and FAQ document. Language Line #: **x-xxx-xxx-xxxx** (Account: **xxxxx**).]

"Hi, this message is for [name]. This is [interviewername] from the Colorado Department of Public Health and Environment. You should have gotten a text message earlier to see if you are interested in participating in a survey about COVID-19 in Colorado. [for Spanish speakers: and that survey link will allow you to choose the Spanish language option]. You can still complete the survey via the link in the text message we previously sent you. We would really appreciate your time to help us with this survey. If you have any questions, you can call us at (**xxx**) **xxx-xxxx** for more information. Thank you for your time, we hope to see your response, and take care."

Interviewer Note: Phone number sending text messages is (**xxx**) **xxx-xxxx**

Check this box if left voicemail or unable to contact control — ☐ Voicemail or unable to contact

Check this box if phone number invalid/disconnected — ☐ Invalid or disconnected

Phone Script:

Hi. This is [interviewername] from the Colorado Department of Public Health. May I please speak with [name]?

The Colorado Department of Public Health and Environment is hoping to collect some information to help us better understand why some individuals are becoming ill with COVID-19 and others are not to help direct our public health response throughout the state. I'm giving you a call because you've been identified as someone who has recently tested negative for COVID-19, I'm wondering if you would be willing to help us with this study by going through a confidential interview that might take 10-15 minutes?

[If they say yes and are curious about what the study is] Thank you, this information will help us better control COVID-19 and direct our public health response so that it meets the needs of communities that are being most affected by COVID-19. Any information you provide us will be confidential and stored in a secure location.

[If they hesitate or say it is not a good time] "Would you be willing to take the survey online on your own time? You should have received a text message with a link, or I can re-send it to you? Is this a good phone number to text, or I can email?" [if yes, insert details below]

What questions or concerns do you have before we continue? If there is anything you don't understand or want me to explain, please let me know at any time during this call.

| | |
|---|---|
| Phone or email for re-sending link: | ______________________ |
| Check this box if they requested that the link be resent | ☐ Requested that link be resent |
| Check this box if they requested that the link be resent in Spanish: | ☐ Requested link resent in Spanish |
| Resent by: | ______________________ |
| Are you willing to participate in this survey and answer all questions as honestly and accurately as possible? | ◯ Yes<br>◯ No |
| Do you prefer proceeding in English or in another language? | ◯ I prefer English.<br>◯ I prefer another language. |
| What language do you prefer? | ______________________ |

Interviewer Note: If control prefers a language other than English, respond with: Thank you for letting me know. We can use an interpreter to help us complete this interview in [language_other]. I'll go ahead and end this call, and call back with an interpreter on the line, does that sound ok?"

## Control Screening Questions

I'll start by asking you some questions about COVID testing.

| | |
|---|---|
| Have you ever tested positive for COVID-19? | ◯ Yes<br>◯ No<br>◯ Unknown |
| Did you receive a COVID vaccine before the date you were tested?<br><br>This includes any dose of vaccine (1 or 2 jabs). | ◯ Yes<br>◯ No<br>◯ Unknown |
| During the 14 days before you were tested, were you living with someone in your household who tested positive for COVID-19?<br><br>Household members are people you live with some or most of the time. This might be roommates, family, or others who share living areas including sleeping, bathroom, and kitchen eating areas. | ◯ Yes<br>◯ No<br>◯ Unknown |
| During the 14 days before you were tested, were you living in a long-term care facility, such as a nursing home (including skilled nursing, hospice care, or long-term acute care)? | ◯ Yes<br>◯ No<br>◯ Unknown |
| What date were you most recently tested for COVID-19?<br><br>This is the date you received the test (had the swab done), not the date you got results. | ______________________ |
| What were the reason(s) you were tested for COVID-19? Select all that apply. | ☐ Had COVID-19-like symptoms<br>☐ Travel<br>☐ Known exposure to person with confirmed or suspected COVID-19<br>☐ Screening as part of job<br>☐ Screening as resident of congregate living facility<br>☐ Was curious or wanted to know status<br>☐ Had some symptoms but not exactly COVID-19-like<br>☐ Doctor/healthcare provider recommended it<br>☐ Other (specify): ______ |
| How many times have you been tested for COVID-19 before this negative test?<br><br>This does not include the current negative test. | ______________________ |
| How many times have you been tested for COVID-19 before this negative test?<br><br>This does not include the current negative test. | ◯ 0 times<br>◯ 1-5 times<br>◯ >5 times |
| Check here if interviewee refused these questions: | ☐ Refused |

## Exposures and Activities

For this next set of questions, it may be helpful for you to have a calendar in front of you in order to review some dates with me. It is really important for us to be as accurate as possible with these dates. I'd like to ask you some questions about what you did in the 14 days before you were tested ([testdate]). Feel free to use your calendar, phone, or social media to help jog your memory about what you did and who you had contact with.

---

Did you visit a healthcare setting in the 14 days before you were tested?

A healthcare setting could include a doctor's or dentist's appointment, hospital visit, or visit for medical care, either if you were a patient or you visited someone else or went to someone else's appointment with them.

- ◯ Yes
- ◯ No
- ◯ Unknown

---

How many times did you visit a healthcare setting in the 14 days before you were tested?

__________________________________

---

As far as you are aware, did you have contact with anyone with confirmed or suspected COVID-19 in the 14 days before you were tested?

Contact is defined as being within 6 feet of an infected person for a total of 15 minutes or more, whether they were wearing a mask or not.

Interviewer note: This is only contact that someone is aware of. Many people may say they do not know if they were in contact with someone who had COVID-19, so an unkonwn response here would be "No".

- ◯ Yes
- ◯ No

---

Describe your contact with the known case of COVID-19: Select all that apply.

Congregate housing refers to housing where individuals are living in close quarters or share common spaces

- ☐ Living in the same household
- ☐ In my house but not LIVING in my house (intimate partner, providing care at home)
- ☐ In a healthcare facility (for example, hospital, clinic, MD office, dialysis)
- ☐ In a long-term care facility (for example, skilled nursing facility, rehabilitation center)
- ☐ In other congregate housing / multi-family dwelling (for example, living in close quarters or sharing common spaces)
- ☐ At a daycare or school
- ☐ At my place of employment
- ☐ At a social event or gathering
- ☐ Place of worship / Religious event
- ☐ Political Rally / Gathering
- ☐ Sports Event
- ☐ Travel
- ☐ Unknown/Unsure
- ☐ Other (specify): ______

| | |
|---|---|
| What is your relationship to the person who had (or may have had) COVID-19? Select all that apply. | ☐ Spouse/Partner<br>☐ Child<br>☐ Parent<br>☐ Other Family<br>☐ Friend<br>☐ Healthcare Worker<br>☐ Co-worker<br>☐ Classmate<br>☐ Roommate / housemate<br>☐ Contact only - no relationship<br>☐ Other (specify): ______ |
| As far as you know, did this person test positive for COVID-19? | ○ Tested positive<br>○ Tested negative or unsure of results<br>○ Unsure if tested<br>○ Not tested |

Now I'd like you to walk me through what in-person activities you did in the 14 days before you were tested ([testdate]). For these questions, do not include activities you did as part of paid work. Interviewer note: These questions are intended to capture high-risk exposures, where masking, distancing, and avoiding congregation outside the household may not have been possible.

| | |
|---|---|
| Did you attend any gatherings in the 14 days before you were tested?<br><br>Interviewer note: A gathering includes any gathering outside the home (in a public or private setting) with members outside of the individual's household. | ○ Yes ○ No ○ Unknown |
| How many gatherings did you attend? | ______________________ |
| Did you go to a restaurant/café/coffee shop in the 14 days before you were tested (do not include takeout, curbside/window pickup or food delivery)? | ○ Yes ○ No ○ Unknown |
| How many times? | ______________________ |
| How many times did you dine indoors? | ______________________ |
| How many times did you dine in a structure outdoors (for example, tents, yurts, igloos, pods, etc.) with people not in your household? | ______________________ |
| How many times did you dine outdoors (open air, not in a structure) with people not in your household? | ______________________ |
| Did you go to a bar or club in the 14 days before you were tested? | ○ Yes ○ No ○ Unknown |
| How many times? | ______________________ |

| | |
|---|---|
| How many times did you go to a bar or club indoors? | __________________ |
| Did you attend any church/religious/spiritual gatherings in the 14 days before you were tested? | ◯ Yes ◯ No ◯ Unknown |
| How many times? | __________________ |
| How many times were you indoors at church/religious/spiritual gatherings? | __________________ |
| Did you go to a gym/fitness center/workout group in the 14 days before you were tested? | ◯ Yes ◯ No ◯ Unknown |
| How many times? | __________________ |
| How many times were you indoors at a gym/fitness center/workout group? | __________________ |
| Did you attend a large sporting event, such as a Rockies game, in the 14 days before you were tested?<br><br>Interviewer note: This includes large, mostly professional sporting events where you pay a fee to enter and attend (as opposed to attending a child or relative's soccer tournament, for example). | ◯ Yes ◯ No ◯ Unknown |
| How many times? | __________________ |
| How many times were you indoors at a sporting event? | __________________ |
| Did you attend a sports practice/game/tournament as a participant or spectator in the 14 days before you were tested? | ◯ Yes ◯ No ◯ Unknown |
| How many times? | __________________ |
| How many times were you indoors at a sports practice/game/tournament? | __________________ |
| Were you a: | ◯ Spectator<br>◯ Participant (including player, coach, referee) |
| Did you participate in any snow sports (skiing, snowboarding) in the 14 days before you were tested? | ◯ Yes ◯ No ◯ Unknown |
| How many times? | __________________ |
| How many times were you at a resort? | __________________ |

| | |
|---|---|
| Did you attend a wedding/funeral/birthday party (or other similar gathering) in the 14 days before you were tested? | ◯ Yes ◯ No ◯ Unknown |
| How many times? | __________________________________ |
| How many times were you indoors at a wedding/funeral/birthday party (or other similar gathering)? | __________________________________ |
| Did you go to a grocery store (not curbside/outside pickup) in the 14 days before you were tested? | ◯ Yes<br>◯ No<br>◯ Unknown |
| How many times? | __________________________________ |
| Did you go to any retail shopping venues other than a grocery store? (not curbside/outside pickup) in the 14 days before you were tested? | ◯ Yes<br>◯ No<br>◯ Unknown |
| How many times? | __________________________________ |
| Did you attend any protests or rallies in the 14 days before you were tested? | ◯ Yes ◯ No ◯ Unknown |
| How many times? | __________________________________ |
| Did you go to a salon, spa, or barber in the 14 days before you were tested?<br><br>Interviewer note: Include hair salons, nail salons, massage, and other similar venues. | ◯ Yes<br>◯ No<br>◯ Unknown |
| How many times? | __________________________________ |
| Did you visit a casino in the 14 days before you were tested? | ◯ Yes<br>◯ No<br>◯ Unknown |
| How many times? | __________________________________ |
| Are there any additional places you went or activities you did outside your home in the 14 days before you were tested ([testdate])? | __________________________________ |
| Check here if interviewee refused these questions: | ☐ Refused |
| Camping/boating/hunting | ◯ Yes ◯ No ◯ Unknown |

How many times did you go camping/boating/hunting in the 14 days before you were tested (only count gatherings on different days)? ______________________

Casino/hotel ◯ Yes ◯ No ◯ Unknown

How many times did you attend a casino/hotel in the 14 days before you were tested (only count visits on different days)? ______________________

Shopping/flea market ◯ Yes ◯ No ◯ Unknown

Interviewer note: "shopping" includes grocery stores (but not curbside pickup).

How many times did you go shopping or to a flea market in the 14 days before you were tested (only count visits on different days)? ______________________

## Employment Information

In this next section, we will talk about where you work, or volunteer. Remember, all of this information will remain confidential.

In the 14 days before you were tested, did you work or volunteer outside of your home?
◯ Yes
◯ No

What kind of industry are you in? Select all that apply.

Interviewer note: This question is designed to capture specific high risk industries, and most industries will not get captured by the close-ended responses. For example, healthcare should only include people working in a healthcare setting that puts them at high risk (ie, patient care), not people working in research or administration.

- ☐ Healthcare
- ☐ Emergency Response (Fire, police, EMS)
- ☐ Grocery store
- ☐ Retail store (not food)
- ☐ Restaurant/Fast Food
- ☐ Construction
- ☐ Transportation
- ☐ Education (Child Care, K-12, or Higher Education Settings)
- ☐ Food manufacturing or meat packing
- ☐ Manufacturing (not food)
- ☐ Hospitality
- ☐ Agriculture
- ☐ Military
- ☐ Other (please specify below)

Please specify other industry ______________________

What is your occupation? ______________________

Check here if interviewee refused these questions: ☐ Refused

## Travel Information

Now I'd like to ask you about travel in the 14 days before you were tested ([testdate]).

| | |
|---|---|
| Did you travel in the 14 days before you were tested? Select all that apply. | ☐ International<br>☐ Domestic (within the U.S.)<br>☐ Within Colorado (Specifically, outside of county of residence for work, shopping, or recreation)<br>☐ None |
| Did you spend time on an airplane or in an airport in the 14 days before you were tested? | ◯ Yes<br>◯ No |
| Did you stay overnight in a hotel in the 14 days before you were tested? | ◯ Yes<br>◯ No |
| Did you use public transportation in the 14 days before you were tested? | ◯ Yes<br>◯ No<br>◯ Unknown |
| How many times did you use public transportation? | __________________________________ |
| When you left your home in the 14 days before you were tested, how often did you wear a face covering or mask when in an indoor space? | ◯ Always<br>◯ Sometimes<br>◯ Rarely<br>◯ Never<br>◯ Unknown |
| When you left your home in the 14 days before you were tested, how often did you wear a face covering or mask when in an outdoor space? | ◯ Always<br>◯ Sometimes<br>◯ Rarely<br>◯ Never<br>◯ Unknown |
| What type of mask did you typically wear? Select all that apply | ☐ Cloth mask<br>☐ Medical mask<br>☐ KN95 mask<br>☐ Gaiter<br>☐ Double mask<br>☐ I do not typically wear a mask |
| Which of the following actions did you use to protect yourself or others from COVID-19 in the 14 days before you were tested? Select all that apply | ☐ Social distancing more than 6 feet away from people when outside your home<br>☐ Stayed home almost the entire time<br>☐ Did not visit family or friends in their homes<br>☐ Did not travel other than local errands<br>☐ Postponed/cancelled non-essential medical appointments, procedures, or surgeries<br>☐ Postponed/cancelled other appointments such as hair or nails<br>☐ Kept others out of your home<br>☐ Increased hand washing or use of hand sanitizer<br>☐ Other ______<br>☐ None of the above |
| Check here if interviewee refused these questions: | ☐ Refused |

| | |
|---|---|
| Did you travel on a cruise ship or vessel as a passenger or crew member? | ◯ Yes<br>◯ No |

## High-Risk Settings

I would like to ask you a few questions about your living conditions.

| | |
|---|---|
| In the 14 days before you were tested, were you living in a group setting?<br><br>For example, a group home, dormitory, migrant worker housing, or any other setting where people live in close quarters? | ◯ Yes<br>◯ No |
| What type of residence or group setting? | ◯ Federal prison<br>◯ State prison<br>◯ Jail<br>◯ Skilled nursing<br>◯ Assisted living<br>◯ Independent living<br>◯ Hospice<br>◯ Long term acute care<br>◯ Psychiatric hospital<br>◯ School or college dorm<br>◯ Group home<br>◯ Facility for developmentally disabled<br>◯ Rehab facility<br>◯ Alcohol/drug abuse treatment center<br>◯ Homeless shelter<br>◯ Correctional - Other<br>◯ Other group living setting |
| Are you currently experiencing homelessness, or were you experiencing homelessness during the 14 days before you were tested? | ◯ Yes<br>◯ No |
| Please provide the total number of people in your household, including yourself.<br><br>Household members are people you live with some or most of the time. They might be roommates, family, or others who share living areas including sleeping, bathroom, and kitchen eating areas. | ______________________ |
| Check here if interviewee refused these questions: | ☐ Refused |

## Clinical Information

Did you experience any of the following symptoms in the 14 days before you were tested ([testdate])?  Interviewer note: If a control reports symptoms related to a chronic condition, ask them if those symptoms changed in the 14 days prior to testing. If the symptom is not new or did not noticably change, do not record it as a symptom.

| | |
|---|---|
| Fever (measured or subjective) | ◯ Yes ◯ No ◯ Unknown |

| | |
|---|---|
| Fever over 100.4 F (38 C) | ◯ Yes ◯ No ◯ Unknown<br>◯ Did not take temperature |
| Chills | ◯ Yes ◯ No ◯ Unknown |
| Sweats (rigors) | ◯ Yes ◯ No ◯ Unknown |
| Dehydration | ◯ Yes ◯ No ◯ Unknown |
| Sore throat | ◯ Yes ◯ No ◯ Unknown |
| Runny nose (rhinorrhea) | ◯ Yes ◯ No ◯ Unknown |
| Stuffy nose (nasal congestion) | ◯ Yes ◯ No ◯ Unknown |
| Loss of sense of smell or taste | ◯ Yes ◯ No ◯ Unknown |
| Cough (new onset or worsening of chronic cough) | ◯ Yes ◯ No ◯ Unknown |
| Shortness of breath (breathlessness, breathing rapidly) | ◯ Yes ◯ No ◯ Unknown |
| Difficulty breathing (unable to take a good breath) | ◯ Yes ◯ No ◯ Unknown |
| Wheezing | ◯ Yes ◯ No ◯ Unknown |
| Chest pain | ◯ Yes ◯ No ◯ Unknown |
| Abdominal (stomach) pain | ◯ Yes ◯ No ◯ Unknown |
| Nausea or vomiting | ◯ Yes ◯ No ◯ Unknown |
| Diarrhea (3 or more loose stools/day) | ◯ Yes ◯ No ◯ Unknown |
| Body aches | ◯ Yes ◯ No ◯ Unknown |
| Headache | ◯ Yes ◯ No ◯ Unknown |
| Fatigue | ◯ Yes ◯ No ◯ Unknown |
| Confusion (altered mental status) | ◯ Yes ◯ No ◯ Unknown |
| Any other symptoms not listed above? | ◯ Yes ◯ No ◯ Unknown |
| Please describe any other symptoms you experienced: | ______________________ |
| Check here if interviewee refused these questions: | ☐ Refused |

## Demographic Information

Next I have some questions to ask about how you identify yourself - you are not required to answer these questions if you prefer not to do so.

What is your month of birth?

- ◯ January
- ◯ February
- ◯ March
- ◯ April,
- ◯ May
- ◯ June
- ◯ July
- ◯ August
- ◯ September
- ◯ October
- ◯ November
- ◯ December

What is your day of birth?

__________________________________

How do you identify your sex?

- ◯ Male
- ◯ Female
- ◯ Female to Male
- ◯ Male to Female
- ◯ Unknown or Other

How would you describe your race? Select all that apply.

- ☐ American Indian or Alaskan Native
- ☐ Asian
- ☐ Black or African American
- ☐ Native Hawaiian or Other Pacific Islander
- ☐ White
- ☐ Other ______
- ☐ Prefer not to answer
- ☐ Unknown

How would you describe your ethnicity?

- ◯ Hispanic or Latino
- ◯ Not Hispanic or Latino
- ◯ Prefer not to answer
- ◯ Unknown

Check here if interviewee refused these questions: ☐ Refused

Thank you for participating in this survey! You are helping Colorado understand more about COVID-19 and reduce illness in our state. If you have any questions, please contact the Project Coordinator at **xxxxx**@state.co.us, call us at (**xxx**) **xxx-xxxx**, or visit https://covid19.colorado.gov/case-control-comparison.

Add any issues or notes your had during this interviewer here:

__________________________________________
